# Clubfoot: Etiology and treatment

**DOI:** 10.4103/0019-5413.38576

**Published:** 2008

**Authors:** Ashish Anand, Debra A Sala

**Affiliations:** Clubfoot Center, Center for Children, NYU Hospital for Joint Diseases, New York, USA

**Keywords:** Clubfoot, French technique, kite technique, Ponseti

## Abstract

Congenital talipes equinovarus is the commonest congenital anomaly with an incidence of one to two per 1000 live births. Over the centuries it has been treated by various modalities, but the dilemma facing the surgeon has been a strong tendency to relapse. With the use of the Ponseti technique, the number of patients who undergo soft tissue release has decreased. This technique probably represents a panacea for the treatment of this unsolved mystery.

## INTRODUCTION

Clubfoot is one of the most common congenital orthopedic anomalies and was described by Hippocrates in the year 400 BC.^1^ However, it still continues to challenge the skills of the pediatric orthopedic surgeon as it has a notorious tendency to relapse, irrespective of whether the foot is treated by conservative or operative means. Part of the reason that the foot relapses is the surgeon's failure to recognize the underlying pathoanatomy. Clubfoot is often automatically assumed to be an equinovarus deformity, however, other permutations and combinations, such as calcaneovalgus, equinovalgus and calcaneovarus, are possible. Out of these four combinations, calcaneovalgus occurs most frequently, followed by equinovarus deformity. In more than 99% of the cases, calcaneovalgus responds to conservative treatment, which involves passive manipulation by the mother and usually does not require casting or operative intervention.^2^

The equinovarus deformity is classified into congenital and acquired. The congenital is further classified into idiopathic and non-idiopathic types. The idiopathic type is typically an isolated skeletal anomaly, usually bilateral, has a higher response rate to conservative treatment and a tendency towards a late recurrence. The causes of the non-idiopathic type include deformity occurring in genetic syndromes, teratologic anomalies, neurological disorders of known (e.g., spina bifida) and unknown etiology and myopathies. The non-idiopathic type is characterized by diametrically opposite deformities in the feet (calcaneovalgus in one foot and equinovarus in the other), presence of other anomalies and a poor response to conservative or operative treatment. Acquired equinovarus has neurogenic causes (e.g, poliomyelitis, meningitis, sciatic nerve damage) and vascular causes (Volkmann Ischemic Paralysis).

This review will concentrate on the treatment of the idiopathic equinovarus deformity, which will be referred to as clubfoot.

## PATHOANATOMY

A postural deformity needs to be distinguished from a true clubfoot. The cause of the postural deformity is the position *in utero* in contrast to the true clubfoot, which has an underlying pathology.^3^ Additionally, the postural condition usually responds to passive manipulation by the mother.

The anatomy was first described by Scarpa^2^ in 1800 and has been subsequently verified by other authors such as Kite and Turco.^4^ According to Scarpa, clubfoot is a congenital talocalcaneonavicular (TCN) joint dislocation, which is the currently accepted view. In contrast, Goldstein believes that the primary abnormality is outward rotation of the talus in the ankle mortise.^4^

The true clubfoot is characterized by equinus, varus, adductus and cavus. The equinus deformity is present at the ankle joint, TCN joint and the forefoot. In the varus component, the hind foot is rotated inwards and this occurs primarily at the TCN joint. The whole of the tarsus, except for the talus, is rotated inward with respect to the lower leg. Since the forefoot follows the hind foot, the medial border of the forefoot faces upward. The adductus deformity takes place at the talonavicular and the anterior subtalar joints. The cavus component involves forefoot plantar flexion, which contributes to the composite equinus.

The pathology of the individual bones contributes to the clubfoot deformity. The multiple abnormalities of the talus include broadening of the anterior part of the trochlea, increased medial deviation of the neck, foreshortening of the neck, absence of the normal constriction of the neck and flattening of the talar head. Additionally, the inferior surface of the talus is characterized by hypoplasia of the posterior concave facet and the three plantar facets of the head appear as a single mass.^5^ The calcaneus is involved in all of the components of the deformity and is grossly normal except that the three facets on the dorsal surface are flattened and the sustentaculum tali is hypoplastic.^6^ The navicular is displaced medially and its proximal concavity is flattened as a result of it having never articulated with the talus.^6^ The cuboid moves medially with the anterior end of the calcaneus and this causes the lateral convexity of the foot.

It is the TCN joint dislocation with the soft tissue contractures around the ankle and TCN joint that maintains this deformity. These contractures involve muscles, tendons, tendon sheaths, ligaments and joint capsules. The contractures are divided into four groups: posterior, medial plantar, subtalar and plantar. The posterior contractures include the tendo Achilles, tibiotalar capsule, talocalcaneal capsule, posterior talofibular ligament and calcaneofibular ligament. The medial plantar contractures involve the talonavicular capsule, deltoid ligament, tibialis posterior tendon and spring ligament. The subtalar contractures include the talocalcaneal interosseus ligament and the bifurcated Y ligament. The plantar contractures are the abductor hallucis, plantar fascia and intrinsic toe flexors.

An important structure, which deserves elaboration, is the Master Knot of Henry, which overlies the navicular tuberosity. It is a fibrous anlage formed by the inter-communication of the tendon sheaths of the flexor digitorum longus and the flexor hallucis longus and should be released at the time of surgery otherwise there is a tendency to recur.^7^

## ETIOLOGY

Numerous etiologies have been proposed, discarded, rediscovered by the next generation and represented. Many theories are in vogue because no single theory adequately explains the erratic response of the clubfoot to treatment. One of the first ones, described by Hippocrates, was the mechanical theory,^4^ which postulates that clubfoot results from an elevated intrauterine pressure during pregnancy. This was disputed because of the absence of increased incidence in an overcrowded uterus (twinning, large babies, hydramnios and primiparous uterus). In the past, a neuromuscular etiology has been proposed based on the histochemical analysis of the clubfeet.^8^ They observed an increase in Type I:II muscle fiber ratio from 1:2 to 7:1, which suggests a possible neural basis. However, Irani^9^ observed no such abnormality.

Several authors have advanced histological theories. Loren *et al*.^10^ have shown that abnormal peroneus brevis histology correlates with higher chances of relapse. A primary germ plasm defect was proposed by Irani.^9^ Defects in the cartilage have been reported by Shapiro and Glimcher^11^. An increased collagen synthesis was found by Ionasescu.^12^ Ippolito and Ponseti^13^ have described the theory of retraction fibrosis of the distal muscles of the calf and supporting connective tissue.

Additionally, anatomical abnormalities have been postulated to explain the occurrence of clubfoot. Ippolito^14^ demonstrated medial angulation of the neck and medial tilting and rotation of the body of talus. Hootnick^15^ and associates described hypoplasia of the anterior tibial artery in patients with clubfeet. Turco^2^ and Porter^3^ have shown anomalous muscles in about 15% of patients with clubfoot.

An alternative theory of arrested fetal development, was proposed by Von Volkmann in 1863 and has subsequently been verified by other authors.^2^ According to this theory, the foot is normally in equinovarus and corrects to a pronated foot at birth. The development of the fetal foot is arrested because of an intrinsic error or an environmental insult, which retards the correction of the physiological position to the normal pronated foot and results in the clubfoot seen at birth.

Studies by Palmer and Davies^4^ have shown that clubfoot is inherited as a polygenic multifactorial trait, which implies that genetic factors do play an important role, but the mode of inheritance is not clear. A higher prevalence of clubfoot was found in children who were born between December and March than at other times of the year.^16^ Edwards *et al*.^17^ propose maternal hyperthermia as an adverse environmental factor in the sensitive period of intrauterine development.

The consensus theory, which incorporates all of the above mentioned theories, probably best explains the occurrence of clubfoot.

## ANTENATAL DIAGNOSIS

With the advent of ultrasound, clubfoot can now be diagnosed at 18-20 weeks of gestation. However, this is only 80% accurate. If the antenatal diagnosis is made at <20 weeks, some authors^18^,^19^ have suggested amniocentesis because of the high incidence (14.2%) of associated genetic anomalies, such as Trisomy18, Larsen's syndrome, neural tube defects and congenital heart defects. Considering the high false positive rate of the ultrasound and the associated risk of fetal loss with amniocentesis, this has not been accepted as the standard of care in the United States.

## CLINICAL FEATURES

Idiopathic clubfoot is characterized by a bean-shaped foot, prominence of the head of the talus, medial plantar cleft, deep posterior cleft, absence of normal creases over the insertion of tendo achilles, calcaneal tuberosity situated at a higher level and atrophy of calf muscles. The three major components of the deformity, that is, equinus, varus and adductus, are obvious on examination. The attitude of the knee is usually flexed, but in cases of neglected clubfoot, the attitude of the knee will be hyperextension. The other parts of the body should be examined to rule out other anomalies. The presence of other anomalies implies a non-idiopathic type of clubfoot, which as previously noted has a poor prognosis.

## RADIOLOGY

X-rays are not routinely ordered at birth as few bones in the foot are ossified. However, if one suspects a teratological etiology, then one should order X-rays immediately to document the same. X-rays, if done at all, are taken at three to four months of age.^20^ The two views utilized are AP and lateral in stress dorsiflexion. The angles that are measured on the AP view are the talocalcaneal angle (normal 30-50 degrees) and the talo-first metatarsal angle (normal 0-10 degrees). The angles that are measured on lateral view are the talocalcaneal angle (normal 30-50 degrees) and the tibiocalcaneal angle (10-20 degrees). In the clubfoot, all of these angles are decreased.

## CLASSIFICATION

Clubfoot has been classified in the past as mild, moderate and severe, but this is considered to be too subjective. Three classification systems that are accepted worldwide are the Dimeglio *et al*.^21^ classification system, Pirani^22^ and International Clubfoot Study Group (ICFSG) classification system. Flynn *et al*.^23^ and Celebi *et al*.^24^ have shown that after an initial learning curve, all three systems had very good interobserver and intraobserver reliability. We will not be discussing the classification here and the reader is referred to the relevant texts for further study.

## TREATMENT

The treatment of clubfoot can be divided into two phases, the pre-Ponseti era and post-Ponseti era. In the pre-Ponseti era, stress was on conservative treatment and followed by operative treatment if the conservative treatment failed. The Ponseti technique is essentially conservative. This does not suggest that in the post-Ponseti era all the other modalities have been abandoned. Other methods, including surgery, are still being followed depending upon individual preferences.

The first non-operative treatment was proposed by Hippocrates in 400 BC when he recommended gentle manipulation followed by splinting.^25^ Plaster casts were used to treat clubfoot when Guerin^26^ introduced the plaster of Paris in 1836. Kite^1^ was the first to recommend gentle manipulation and cast immobilization.

At the annual meeting of the American Academy of Orthopedic Surgeons in 2002, Cummings^20^ stated, “There are as many techniques for manipulative treatment of congenital clubfoot as there are authors who write about clubfoot”. To circumvent this problem, International Clubfoot Study Group, established in 2003, has approved Kite's, Ponseti's and Bensahel's techniques as the standardized conservative regimes for the treatment of clubfoot all over the world.^27^

### Kite's technique

In Kite's method, the manipulation can be started soon after birth. It was derived from the concept of three-point pressure, such as used in the bending of a wire. The fulcrum is the calcaneocuboid joint. The forefoot is grasped and distracted while the other hand holds the heel. Applying counterpressure over the calcaneocuboid joint the navicular is pushed laterally. The heel is everted as the foot is abducted. This is followed by the application of a slipper cast, which is extended to below the knee with the foot everted with gentle external rotation. Afterwards, the foot is pushed into dorsiflexion to correct the equinus once the adductus and varus are corrected. The casts are changed every week. Following full correction, the feet are placed in a Denis Browne Bar. The success rate varies from a high of 90% found by Kite to a low of 19% by Fripp and Shaw.^2^ According to Ponseti,^28^ the average number of casts required for correction by this technique is 20.4.

### Ponseti technique

Ponseti had been reporting consistent results since 1950, but it is only recently that he has been given due recognition. His technique is based on the solid understanding of the pathoanatomy of clubfoot. According to Ponseti, the clubfoot usually recurs until four years of age and parents should be warned of this possibility. Ponseti suggests two reasons for the poor results found with Kite's technique. First, the use of the calcaneocuboid joint as the fulcrum blocks the abduction of the calcaneus and thereby prevents eversion of the calcaneus. Secondly, pronation of the forefoot to correct the cavus actually worsens the cavus. A recent study by Frick^29^ highlights the importance of correction of the supination. Based on laboratory studies, Ponseti^28^ has shown that the calcaneus everts only when it is fully abducted.

In Ponseti's technique, the first two casts are applied with the forefoot supinated so as to bring it into alignment with the hind foot.^30^ The third cast is applied with the forefoot abducted and simultaneous counterpressure over the head of talus. In the fourth cast, the forefoot is further abducted. Prior to the fifth cast, the degree of dorsiflexion is assessed and if dorsiflexion is not possible beyond neutral, then a percutaneous Achilles tenotomy is required. The tenotomy, if required, is done under local anesthesia as an outpatient procedure. The casts before the tenotomy are changed at weekly intervals while the cast after the tenotomy is removed at the end of three weeks. The average number of casts with the Ponseti technique is only 5.4 compared to the 20 casts with Kite's technique and this results in saving time and money for the patient.^31^ Following the removal of the last cast, irrespective of whether a tenotomy is done or not, the patient is placed in a modified Foot Abduction Orthosis (FAO), which is used for 23 h a day in the initial four months and then subsequently for nighttime for three years.^32^ According to Ponseti, a tenotomy is required in 70% of the cases.^28^ In a study by Scher *et al*.,^33^ children with clubfeet who have an initial score of ≥5.0 by the Pirani system or are rated as Grade IV feet by the Dimeglio system are very likely to need a tenotomy.

Clubfoot has a strong tendency to relapse until four years of age and this is attributed to the original pathology. Relapses decrease after age four because the pathology that causes clubfoot ceases to exist. According to Ponseti,^34^ 50% of the relapses occurred between 10 months to five years and this was irrespective of the degree of correction that was obtained after casting. The single most important factor that predicts recurrence is noncompliance with the FAO and the recurrence rate could be reduced to 10% if the patient was compliant with the FAO.^34^ In a recent study by Thacker *et al*.^35^ the feet of the patients who were compliant with the FAO maintained their correction better than those who were not compliant. In a comparison of two groups (34 each), one treated by Ponseti technique and the other by Kite's technique, Herzenberg *et al*.^36^ used postero medial release (PMR) as the end point. For the Ponseti technique group, only one patient needed PMR while in the other group, 32 required PMR. Ninety-one per cent of the patients treated by the Ponseti technique required tenotomy. Lehman *et al*.^37^ have shown excellent early results with the Ponseti technique and according to them good results were possible if casting was begun prior to seven months of age and the patient was compliant with the FAO. Dobbs *et al*.^38^ have reported that noncompliance with the FAO and the educational level of the parents (high-school education or less) are significant risk factors, which predict the increased possibility of recurrence after correction with the Ponseti method. The identification of patients who are at risk for recurrence may allow intervention to improve the compliance of the parents with regard to the use of the FAO and as a result, improve outcome. Probably, the most extensive review and follow-up of the Ponseti technique has been reported by Dobbs *et al*.,^39^ in their evaluation of Ponseti's patients who were treated 25-42 years ago. They found that the corrected clubfeet were less supple and showed no differences in terms of function and performance compared to the normal population. Tibialis anterior tendon transfers were required in 50% of the patients. According to Ponseti,^33^ this should be considered part of the technique and not as a separate operative procedure. In a recent study from Israel, Segev *et al*.^40^ reported excellent results in 94% of the cases with the Ponseti technique.

### French technique

This technique, also known as the Functional method, was introduced in France in the 1970s by Masse and Bensahel,^41^ but it was not until early 1980 that results were available in the English literature. It involved daily manipulation of the child's clubfoot by the physical therapist for 30 min. This was followed by stimulation of the muscles around the foot, especially the peroneal muscles, to maintain the reduction achieved by the passive manipulation and then, adhesive strapping was applied. The daily treatments were continued for approximately two months and then reduced to three sessions per week for an additional six months. Taping was continued until the patient was ambulatory. After ambulation was achieved, a nighttime splint was introduced and used for an additional two to three years. Initially, good results were seen in 50% of the patients and in the remaining cases, the surgery that was required was only a posterior release. The disadvantages of this method were that it involved daily hospital visits, depended on the manipulation skills of the physical therapist and was costly in the long run.^42^ This method was subsequently modified to include placement in a continuous passive motion (CPM) machine for six to eight hours after passive manipulation by the physical therapist and adhesive strapping of the feet. The addition of the CPM machine resulted in fewer patients needing surgery and a less radical procedure for those who required surgery. The success rate was reported to be close to 68%.^43^ With further experience in the use of the CPM machine, the success rate increased to 88%.^43^ This method is not very popular in the United States. In one of the few American studies, Richards *et al*.^44^ reported a success rate of only 44%, but this was without the use of the CPM machine. With the addition of the CPM machine, the success rate went up to 60%.^45^ In a recent study by the same authors,^46^ 42% of the feet did not require surgery to achieve a plantigrade position, 9% needed heel cord tenotomies, 29% needed posterior releases and 20% needed comprehensive PMRs.

### Operative treatment

The list of operative procedures is endless as no single procedure gives a long-lasting correction. The first operative procedure, posterior release, was described by Phelps^2^ in 1891. The PMR procedure, which was introduced by Turco^2^ (1980), is basically a modification of the earlier procedures elaborated by Phelps, Codvilla (1906), Brockman (1937) and Bost (1960).^2^ The rationale behind Turco's PMR was that the deformity is due to the congenital subluxation of the TCN joint, the correction of the abnormal tarsal relationship is prevented by rigid pathologic soft tissue contractures and the correction of any single component of the deformity is impossible while simultaneously eliminating the others. The two prerequisites for lasting correction are that complete correction of all components must be obtained and this correction must be maintained while the tarsal bones remodel.

The optimal age for surgical intervention has always been controversial. Turco recommends surgery at around one year of age while Osterman and Merikanto^47^ recommend surgery at the earlier age of three to six months to utilize the remodeling potential of the foot. However, Danglemajor^48^ advises deferring surgery until one year of age as surgery done earlier has a failure rate close to 65%. Also, the average number of operations per foot was 2.9 to achieve a full correction at skeletal maturity with earlier surgery. In addition to finding a higher incidence of failure, Turco reported the disadvantages of early surgery to be difficulty in the identification of the anatomical structures and in the handling of the small cartilaginous bones when operating on a small foot. Furthermore, when the pins are removed from the talonavicular bones and talocalcaneal bones after a PMR, it is hard to hold the small foot in plaster. Importantly, delaying surgery minimizes the possibility of operating on an unrecognized neuromuscular deformity. One major benefit of operating close to the age of walking is that it takes advantage of the normal physiological stimulus of weight-bearing for remodeling. Turco's procedure was used with impunity in the 1980s with the average failure rate of 25% being reported by Turco himself. Failure rates, ranging from 13 to 50%, were found by Crawford *et al*.^49^ and Vizekelety *et al*.^50^

McKay *et al*.^51^ and Herzenberg *et al*.^52^ have shown that the presence of an internal rotation deformity of the calcaneus cannot be adequately corrected by a PMR alone. They proposed that beyond 18 months of age PMR should be combined with posterolateral release. This can either be done using a single incision of Cinncinnati or Carrolls two incision technique.^53^ The disadvantage of Mckay's procedure is that it results in overcorrection with the heel being placed in valgus in 8-20% of the feet.^54^ Neglected clubfoot is unheard of in the west, however, there have been limited reported series in other parts of the world. The usual protocol that has been followed for the management of such type of feet is surgical either by open surgery^55^,^56^ as described above or by the use of external fixators such as Iliazarov's^57^ and Joshi's External stabilizing system (JESS)^58^ fixators. Casting is done to maintain correction after the fixators are removed. The success rate of correction varies from 77 to 90%.

As can be seen from above, surgical intervention is often followed by complications, residual deformities or recurrence, which require further surgery. The discussion of these surgeries is beyond the context of this article and the reader is referred to specific text for the same.

## SUMMARY

Clubfoot is an enigmatic condition because it can make the best orthopedic surgeons eat humble pie. Treatment over time has varied. Initially, it was Kite's technique which gave excellent results. However, since his results were not reproducible, this was replaced by conservative treatment and/or operative treatment. The dilemma faced by the surgeon was that even after surgery the clubfoot recurs and results in more surgeries and morbidity.

Ever since the introduction of the Ponseti technique, the number of cases requiring soft tissue release has drastically decreased. However, one should remember that the Ponseti technique results are good only if it is followed in its totality including compliance with the FAO. Only time will tell if Ponseti's technique is the answer to this unsolved mystery.
